# PROTOCOL: Evidence‐based frameworks used to promote self‐employment with persons with disabilities: A scoping review

**DOI:** 10.1002/cl2.1350

**Published:** 2023-08-15

**Authors:** Luther Lebogang Monareng, Shaheed Mogammad Soeker, Deshini Naidoo

**Affiliations:** ^1^ Occupational Therapy Department University of KwaZulu Natal Durban South Africa; ^2^ Occupational Therapy Department University of the Western Cape Cape Town South Africa

## Abstract

**Aim:**

This scoping review aims to highlight literature on self‐employment evidence‐based frameworks used to promote self‐employment among persons with disabilities. This will be achieved by answering this research question, *what evidence‐based self‐employment frameworks have been successfully used to promote self‐employment among persons with disabilities?*

**Methods:**

To answer the research question, the steps followed will be based on the Preferred Reporting Items for Systematic Review and Meta‐Analysis (PRISMA). Arksey and O'Malley's sequential stages will be used to guide the scoping review process. An experienced librarian and a second reviewer will assist with this review. A third reviewer will only be involved, when necessary, for example, to help resolve decision conflicts between reviewers one and two.

**Discussion:**

This section will discuss the data collected. In line with Tricco et al., this scoping review envisages outlining existing literature, identifying gaps and informing future research in the field of self‐employment for persons with disabilities. *What evidence‐based self‐employment frameworks have been successfully used to promote self‐employment among persons with disabilities?* Is a question that this scoping review seeks to answer. Thus, this research has the potential to add to knowledge and inform or stimulate further research.

## Key terms

- Evidence‐based framework: In this research, an evidence‐based framework is a set of intervention strategies and information that is based on empirical data and previous experience to guide or facilitate self‐employment with persons with disability. These could be, for example, education and/or training programmes, guidelines, models, or processes.
- Self‐employment: This is the act of being self‐employed. A self‐employed person is ‘an individual who is working for themselves, being directly or indirectly involved in running a successful and profitable small business or microenterprise to earn an income or generate a salary, instead of being employed by another person or an employer’ (Monareng et al., 2021, p. 75).
- Persons with disabilities: These ‘include those who have long‐term physical, mental, intellectual or sensory impairments which in interaction with various barriers may hinder their full and effective participation in society on an equal basis with others’ (United Nations, 2007, p. 4).

## 1 INTRODUCTION

Work is one of the major occupations for adults as it brings about a sense of meaning and purpose and in most cases, it generates income. Vulnerable groups such as persons with disabilities benefit from work as they become active participants in society and realise an increase in social cohesion (American Occupational Therapy Association, 2020; Buckup, 2009; Unger, 2002). Persons with disabilities ‘include those who have long‐term physical, mental, intellectual or sensory impairments which in interaction with various barriers may hinder their full and effective participation in society on an equal basis with others’ (United Nations, 2007, p. 4). Tarvainen (2021) reported that work provides a sense of belonging in the community; this is highlighted by the experiences of participants with disabilities who felt lonely and isolated before work. Therefore, this may imply that those without work may be negatively affected. For instance, they may be subjected to poverty and be dependent on their governments for social support for their survival and livelihood as in countries like South Africa. Poverty has the potential to harm individuals' sense of identity as goal‐directed beings. The importance of work is further confirmed by its inclusion in the United Nation's (UN) 17 sustainable development goals (SDG), that is, decent work and economic growth (United Nations Development Programme, 2015).

People with disabilities make up 15% of the world's population and their rate of unemployment is generally higher than that of their able‐bodied counterparts, that is, there is a 60%–90% unemployment rate among people with disabilities (World Health Organization, 2011). Factors such as the COVID‐19 pandemic perpetuated the plight of unemployment globally which had already been a crisis especially in low to middle‐income countries (LMICs). In an LMIC like South Africa, the statistics on unemployment have recorded an all‐time high of 33.9% (Statssa, 2022). These suggest that non‐disabled individuals will continue to compete for scarce jobs leaving vulnerable groups like persons with disabilities without viable opportunities.

In countries, such as South Africa, the current unemployment crisis has been attributed to its history or inherited legacy created by colonialism and apartheid. Post‐apartheid, the previously oppressed and disadvantaged groups (persons with disabilities included) are observed to be less trained, with limited education, occupying manual labour jobs, and with limited chances of being employed in the already struggling formal labour sector (Gamieldien & van Niekerk, 2017; Phatlane, 2006; Westaway, 2012). As a result, these disadvantaged groups are affected more by unemployment and may benefit from exploring alternative sources of income such as self‐employment to be independent and lead meaningful lives. Self‐employment is the act of being self‐employed. A self‐employed person is ‘an individual who is working for themselves, being directly or indirectly involved in running a successful and profitable small business or microenterprise to earn an income or generate a salary, instead of being employed by another person or an employer’. Monareng et al. (2021, p. 75).

Pagán (2009), highlighted that having a disability increases one's probability of exploring self‐employment as there are benefits such as flexible working hours, it is accommodative of persons with disabilities' impairment and increased levels of satisfaction. Therefore, there should be a shift towards investing more in self‐employment for persons with disabilities, for example, by funding and ensuring that persons with disabilities in self‐employment maintain their social benefits such as grants (Pagán, 2009). Governments' practice seems to be prioritising conventional work, thus maintaining the status quo in terms of injustices associated with it such as discrimination of persons with disabilities at work (Pagán, 2009). As part of the South African government's strategies to reduce unemployment, citizens are urged to explore self‐employment as a source of income or an alternative for fighting the unbearable effects of the global unemployment issue (Bendile, 2016).

The issue of high unemployment rates which affect vulnerable groups, such as persons with disabilities, is a core problem of this research.

Professionals such as occupational therapists possess competencies, knowledge, and skills to facilitate successful participation in occupation, including meaningful work for persons with disabilities. Although a South African study indicated that occupational therapists perceive that they have a role to play in encouraging self‐employment for persons with disabilities, they reported that they do not know how to actively participate in this field (Monareng et al., 2018). The South African‐based occupational therapists also reported that both their undergraduate and postgraduate qualifications did not equip them to be active role players in the field of self‐employment for persons with disabilities (Monareng et al., 2018). Although the role that other professionals play is acknowledged, there is a need for the profession of occupational therapy to contribute towards enhancing persons with disabilities' entrepreneurship skills and presenting them with an alternative option for a source of income.

Occupational therapists have the competencies, knowledge and skills in enabling persons with disabilities to regain their premorbid levels of function in daily activities but no literature outlines an evidence‐based framework to guide them in playing an active role in the field of self‐employment. In this research, an evidence‐based framework considers empirical data from education and/or training programmes, guidelines, intervention strategies, models; processes, strategies, and theories. For these to be considered successful, they must have been proven to yield results for persons with disabilities in self‐employment, that is, they must be tried and tested, they cannot merely be recommendations or proposed solutions.

According to the researchers' knowledge, preliminary literature review and a scoping review pilot search, there are studies conducted on the subject of self‐employment and persons with disabilities. However, what is not apparent, is a study focusing on these three subjects altogether, that is, self‐employment, persons with disabilities, and an evidence‐based framework. This includes professions that have a role to play in work, such professions entail, but are not limited to, social work, industrial psychology, and occupational therapy. The available studies provide the status quo and recommendations instead of tried and tested solutions (Blanck et al., 1999; Coetzee et al., 2011; International Labour Organisation Disability Programme, 2002; Rumrill et al., 2000). This warrants a detailed scoping review to map data on this topic.

The dearth of a contextually relevant evidence‐based framework on self‐employment for professions such as occupational therapy on the African continent may be linked to the Western origin of the Occupational Therapy profession (Bührmann & Bührmann, 1984). This calls for urgent research on an evidence‐based framework that guides occupational therapists in the field of self‐employment to be developed. The framework has the potential to assist them to play an active role in self‐employment for persons with disabilities.

This scoping review aims to highlight literature on self‐employment evidence‐based frameworks used to promote self‐employment among persons with disabilities. This will be achieved by answering this research question, *what evidence‐based self‐employment frameworks have been successfully used to promote self‐employment among persons with disabilities?* To answer this research question, the objectives will be to identify and describe:

i. Existing self‐employment evidence‐based frameworks used with persons with disabilities.
ii. Barriers and facilitators in using a self‐employment evidence‐based framework with persons with disabilities.
iii. Types of successful self‐employment opportunities (small businesses) that persons with disabilities engage in.

## 2 METHODS

### 2.1 Design

A scoping review is explorative and helps synthesise available literature to provide more insight into a topic and pave a platform for future research (Arksey & O'Malley, 2005). This is in line with the aim of this research, as a result, a scoping review will be used.

**Figure 1:**
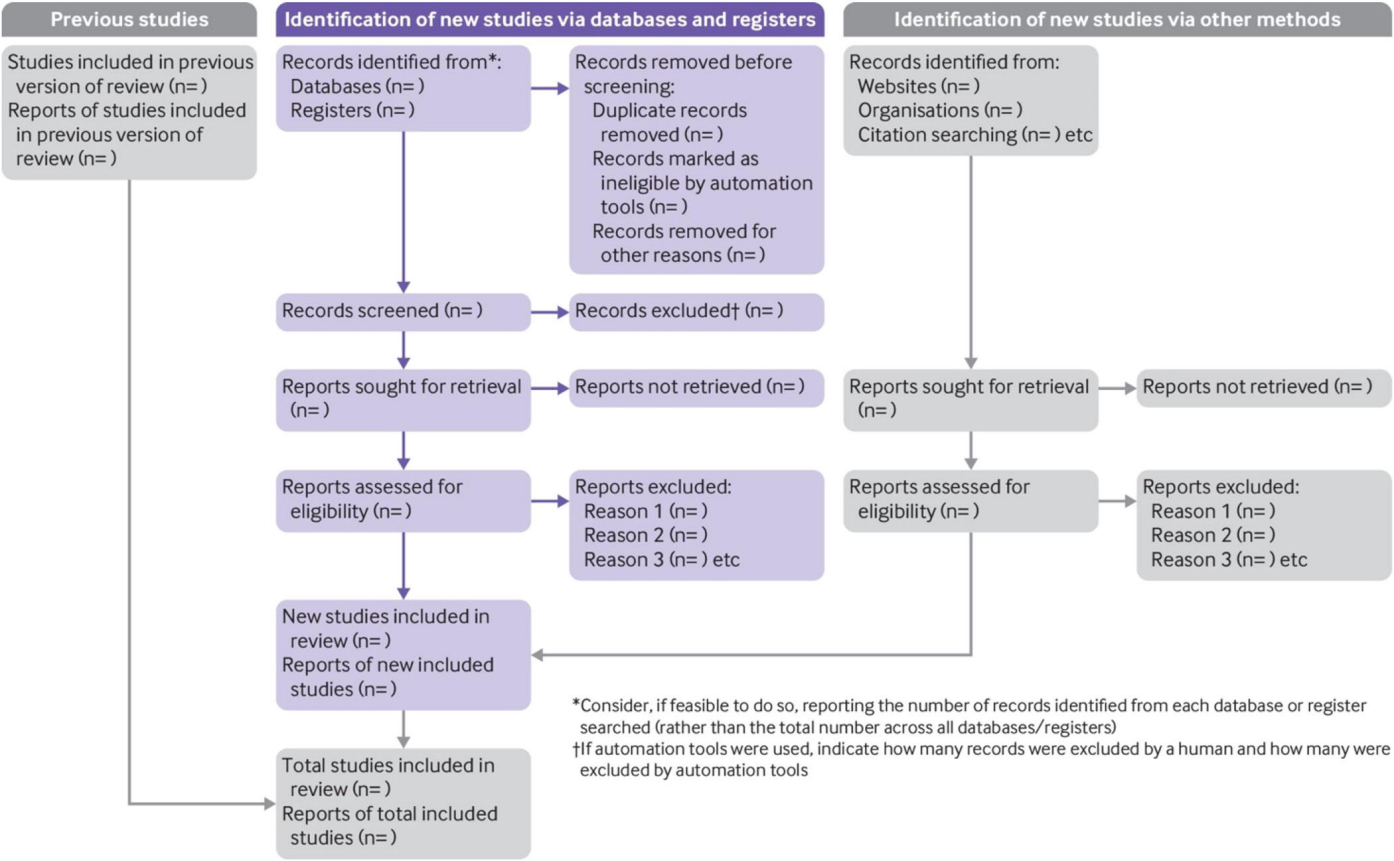
PRISMA 2020 flow diagram template (Page, 2021).

To increase methodological reproducibility and transparency, the process and steps followed will be based on the Preferred Reporting Items for Systematic Review and Meta‐Analysis (PRISMA) (Page, 2021). Table 1 below shows a checklist that will be used to ensure all scoping review aspects are covered. Refer to Figure 1 which displays the PRISMA 2020 flow diagram template (Page, 2021). Arksey and O'Malley's (2005) sequential stages will be used to guide the scoping review process, that is,

**Table 1:** Preferred reporting items for systematic reviews and meta‐analyses extension for scoping reviews (PRISMA‐ScR) checklist (Tricco et al., 2018).

Section	Item	PRISMA‐ScR checklist item	Reported on page #
Title			
Title	1	Identify the report as a scoping review.	1
Abstract			
Structured summary	2	Provide a structured summary that includes (as applicable): background, objectives, eligibility criteria, sources of evidence, charting methods, results, and conclusions that relate to the review questions and objectives.	3–4
Introduction			
Rationale	3	Describe the rationale for the review in the context of what is already known. Explain why the review questions/objectives lend themselves to a scoping review approach.	5–7
Objectives	4	Provide an explicit statement of the questions and objectives being addressed with reference to their key elements (e.g., population or participants, concepts, and context) or other relevant key elements used to conceptualise the review questions and/or objectives.	7
Methods			
Protocol and registration	5	Indicate whether a review protocol exists; state if and where it can be accessed (e.g., a Web address); and if available, provide registration information, including the registration number.	Not applicable
Eligibility criteria	6	Specify characteristics of the sources of evidence used as eligibility criteria (e.g., years considered, language, and publication status), and provide a rationale.	15
Information sources^a^	7	Describe all information sources in the search (e.g., databases with dates of coverage and contact with authors to identify additional sources), as well as the date the most recent search was executed.	14
Search	8	Present the full electronic search strategy for at least 1 database, including any limits used, such that it could be repeated.	13–14
Selection of sources of evidence^b^	9	State the process for selecting sources of evidence (i.e., screening and eligibility) included in the scoping review.	15
Data charting process^c^	10	Describe the methods of charting data from the included sources of evidence (e.g., calibrated forms or forms that have been tested by the team before their use, and whether data charting was done independently or in duplicate) and any processes for obtaining and confirming data from investigators.	16
Data items	11	List and define all variables for which data were sought and any assumptions and simplifications made.	18
Critical appraisal of individual sources of evidence^d^	12	If done, provide a rationale for conducting a critical appraisal of included sources of evidence; describe the methods used and how this information was used in any data synthesis (if appropriate).	Not applicable
Synthesis of results	13	Describe the methods of handling and summarising the data that were charted.	18
Results			
Selection of sources of evidence	14	Give numbers of sources of evidence screened, assessed for eligibility, and included in the review, with reasons for exclusions at each stage, ideally using a flow diagram.	Not applicable
Characteristics of sources of evidence	15	For each source of evidence, present characteristics for which data were charted and provide the citations.	Not applicable
Critical appraisal within sources of evidence	16	If done, present data on critical appraisal of included sources of evidence (see item 12).	Not applicable
Results of individual sources of evidence	17	For each included source of evidence, present the relevant data that were charted that relate to the review questions and objectives.	Not applicable
Synthesis of results	18	Summarise and/or present the charting results as they relate to the review questions and objectives.	Not applicable
Discussion			
Summary of evidence	19	Summarise the main results (including an overview of concepts, themes, and types of evidence available), link to the review questions and objectives, and consider the relevance to key groups.	19
Limitations	20	Discuss the limitations of the scoping review process.	20
Conclusions	21	Provide a general interpretation of the results with respect to the review questions and objectives, as well as potential implications and/or next steps.	Not applicable
Funding			
Funding	22	Describe sources of funding for the included sources of evidence, as well as sources of funding for the scoping review. Describe the role of the funders of the scoping review.	21

Abbreviations: JBI, Joanna Briggs Institute; PRISMA‐ScR, Preferred Reporting Items for Systematic reviews and Meta‐Analyses extension for Scoping Reviews.

^a^ Where *sources of evidence* (see second footnote) are compiled from, such as bibliographic databases, social media platforms, and websites.

^b^ A more inclusive/heterogeneous term used to account for the different types of evidence or data sources (e.g., quantitative and/or qualitative research, expert opinion, and policy documents) that may be eligible in a scoping review as opposed to only studies. This is not to be confused with *information sources* (see first footnote).

^c^ The frameworks by Arksey and O'Malley (6) and Levac and colleagues (7) and the JBI guidance (4, 5) refer to the process of data extraction in a scoping review as data charting.

^d^ The process of systematically examining research evidence to assess its validity, results, and relevance before using it to inform a decision. This term is used for items 12 and 19 instead of ‘risk of bias’ (which is more applicable to systematic reviews of interventions) to include and acknowledge the various sources of evidence that may be used in a scoping review (e.g., quantitative and/or qualitative research, expert opinion, and policy document).

Stage 1: Identifying the research question

Stage 2: Identifying relevant studies

Stage 3: Study selection

Stage 4: Charting the data

Stage 5: Collating, summarising, and reporting the results

**Stage 1: Identifying the research question**

The scoping review initial stage entails identifying the research question while maintaining a wide approach (Arksey & O'Malley, 2005). Our research question is *what evidence‐based self‐employment frameworks have been successfully used to promote self‐employment among persons with disabilities?* This was informed by the aim of this research which entails highlighting literature on self‐employment evidence‐based frameworks used to promote self‐employment among persons with disabilities.

**Stage 2: Identifying relevant studies**

Stage 2 outlines the strategy and sources that will be used to identify relevant studies (Arksey & O'Malley, 2005). Services from an experienced librarian from the University of KwaZulu Natal's College of Health Sciences will be sought to help identify relevant studies. Guided by this study's eligibility criteria, the search will be conducted using published and grey literature.

The EBSCOhost and Scopus platforms, respectively, will be used to search for published studies. Databases that will be searched through EBSCOhost will include but are not limited to, Social Sciences, Health Professions, Psychology, Nursing, Multidisciplinary, and Business. Through Scopus, the following databases are some that will be searched, Academic Search Complete, APA PsycInfo, Business Source Complete, GreenFILE, and Open Dissertations.

For grey literature, platforms that will be searched include, but are not limited to, Google Scholar, International Labour Organization (ILO), ResearchGate, The Organization for Economic Cooperation and Development (OECD), The World Bank, World Health Organization, and the University of Kwa‐Zulu Natal's online library (iCatalogue).

For more breadth, other avenues that could be explored are consulting with experts, authors, hand‐searching journals, and citations. To ensure that published data is up to date, relevant databases will be searched again should the review be published after 12 months from the initial search date. New and relevant data will be incorporated before the publication of the review (Tricco et al., 2018).

A preliminary search (pilot) was conducted to determine the feasibility of this topic. Refer to Table 2 which shows an example of a search strategy from EBSCOhost. The key terms and corresponding alternative terms used are in line with this research's focus, that is, evidence‐based framework, self‐employment, and persons with disabilities. The search will be refined and adapted per platform when necessary to increase the chances of yielding more relevant results. This will be achieved by using, for example, Boolean operators (AND, NOT and OR) (Arksey & O'Malley, 2005). Other search strategies that will be considered are the usage of truncations with words that may have multiple ends such as entrepreneur* to cover words such as entrepreneurial and entrepreneurship. Moreover, introducing relevant profession names to the search terms could be considered, for example, occupational therapy, social work and industrial psychology (The Joanna Briggs Institute, 2015; Tricco et al., 2018).

**Table 2:** Search strategy example from EBSCOhost.

#	S1
Query	(self AND employ* OR entrepreneur* OR business* OR enterprise OR sme*) AND (framework OR model OR strateg* OR program* OR system OR process OR train* OR method OR technique* OR tool* OR guideline OR approach OR practice* OR intervention) AND (persons AND disabil* OR persons AND pwd OR people AND disabil*)
Limiters/Expanders	Limiters—Full Text; References Available; Peer Reviewed; Hidden NetLibrary Holdings
	Expanders—Apply equivalent subjects
	Search modes—Boolean/Phrase
Last Run Avia	Interface—EBSCOhost Research Databases
	Search Screen—Advanced Search
	Database—Academic Search Complete; APA PsycInfo; Atla Religion Database with AtlaSerials; Audiobook Collection (EBSCOhost); Business Source Complete; CAB Abstracts; eBook Collection (EBSCOhost); eBook Open Access (OA) Collection (EBSCOhost); ERIC;GreenFILE; Health Source—Consumer Edition; Health Source: Nursing/Academic Edition; Index to Legal Periodicals Retrospective: 1908–1981 (H.W. Wilson); MasterFILE Premier; MasterFILE Reference eBook Collection; MathSciNet via EBSCOhost; MEDLINE with Full Text; Newspaper Source; OpenDissertations; Regional Business News
Results	1151

A detailed paper trail of databases searched will be kept allowing replication of this research by others. Such a paper trail will entail, but will not be limited to, the platform searched, who conducted the search, search number and date, search strategy, filter used, and the number of studies identified (Tricco et al., 2016). Refer to Table 3 for the database search trail.

**Table 3:** Database search trail.

Platform searched	Who conducted the search	Search number and date	Search strategy	Filter used	Number of studies identified

**Stage 3: Study selection**

Stage 3 entails setting the parameters of what the study will include and exclude, respectively. These parameters will be refined as the reviewers gain familiarity with the topic (Pagán, 2009). The studies that will be included (eligible) must meet the criteria below, that is, those that do not meet the criteria will be excluded:

i. *Primary studies*: Broad and open to all disciplines, that is, the multidisciplinary team such as occupational therapy, education, business and commerce, social work and industrial psychology.
ii. *Concepts*: The evidence‐based framework must be on self‐employment, and with persons with disabilities. The framework must have been tested and the article should report on the empirical data from the test, that is, it must show how it was developed and facilitate self‐employment with persons with disabilities. See Table 2 for corresponding alternative terms. Exclude if offering guidelines and recommendations without testing the framework.
iii. *Context*: All countries
iv. *Language*: English
v. *Type of source*: Online (electronic) sources that are full text with references

Two independent reviewers will be involved, and they will commence by searching the databases using the search strategy in Table 2. The search results (citations) from each database will be exported to a reference manager software (Mendeley) where duplicates will be removed. After removing duplicates, the citations will be exported to a research collaboration tool (Rayyan) for screening the keywords in the title, abstract and index terms. Selection of suitable studies will be done independently and will be guided by the inclusion criteria where reviewers will select ‘Include’, ‘Exclude’ or ‘Maybe’ to indicate their answer. Reasons, especially for excluded studies, will be documented (Arksey and O'Malley, 2005; Tricco et al., 2018).

The reference lists of all suitable (included) studies will be screened to locate additional studies until the saturation point is reached, that is, when no new sources are identified (Arksey & O'Malley, 2005). A full‐text review of all suitable studies will follow to decide which article will be included in the scoping review. The full‐text review step will be conducted independently by two independent reviewers followed by a comparison of findings. Discrepancies between the reviewers will be recorded and resolved through discussions and consensus. A third reviewer will only be involved, when necessary, for example, to resolve decision conflicts between reviewers one and two (The Joanna Briggs Institute, 2015; Tricco et al., 2018).

Although not a requirement for a scoping review, the reviewers will consider assessing the quality of the included articles using the Critical Appraisal Skills Programme (CASP) (Brice, 2021).

**Stage 4: Charting the data**

Data charting will be an iterative process which entails sifting, charting, and sorting. Sift means being clear about what one is looking for, charting entails what exactly one intends to chart to help answer the research question while sorting looks at how to best represent the findings, which may be numerical or narrative (Arksey & O'Malley, 2005; Ritchie & Spencer, 2002).

Using an electronic Microsoft Excel data charting form, data will be extracted and charted independently by two reviewers. The data charting form is aligned with the research topic, question, and objectives of this research. Refer to Table 4 for a data charting form overview. The table was developed by the main reviewer and will be independently reviewed by the second reviewer and then the third reviewer. The form will be amended when necessary. A minimum of two eligible studies at a time will be charted on the form. Discussions will follow immediately to resolve inconsistencies and consensus. A third reviewer will only be involved when necessary (Tricco et al., 2018).

**Table 4:** Data charting form—an overview.

Study characteristics	Objective 1	Objective 2	Objective 3
Author/s (year of publication)	Terms ‐ Tool description term ‐ Targeted end‐user and/or population description term ‐ Self‐employment alternative terms	Barriers/challenges/gaps associated with the tool	Types of successful self‐employment opportunities/small businesses e.g., Car wash, Hair salon
Continent & Country—World Bank Classification by Income	Stakeholders involved and their roles		
Study title	Tool brief description, mention e.g., ‐ Tool name ‐ Aim/purpose/objectives/focus ‐ Stakeholders involved and their roles ‐ The year it started ‐ How was the tool proven to yield results or if it was a success ‐ Conducted in‐person/online/hybrid ‐ Duration/frequency (weeks/months) ‐ If there is a clear process or steps followed, if YES, list them ‐ Other (specify): checklist and forms availability	Facilitators/enablers associated with the tool	Category of successful self‐employment opportunities/small businesses
Method/study design (sample size number)	Process or steps followed		

**Stage 5: Collating, summarising, and reporting the results**

Stage 5 entails collating, summarising, and reporting the results. A narrative report will be produced by summarising charted data from stage 4. Study characteristics will be presented and analysed descriptively whereas the three objectives will be analysed using a matrix and themes. Emerging themes and existing gaps will be highlighted. The themes will be in line with the title, question, and objectives of this research. Gaps regarding published literature will be identified and used to inform potential future research (Arksey & O'Malley, 2005).

### 2.2 Data management

These three platforms will be used to manage data, that is, Mendeley, Rayyan, University of Kwa‐Zulu Natal's online repository. Mendeley is a free reference manager software. Rayyan is a research collaboration tool, and its functions include but are not limited to, allowing multiple reviewers to simultaneously screen or review the same study online, add notes (e.g., reason for excluding a study) and it is compatible with Mendeley. Rayyan has an individual basic plan that is free. The University of Kwa‐Zulu Natal's repository will be used to store data for future use.

People who will have access to this data (during and post this research) are the main reviewer, the second reviewer, and two supervisors. Data will be available for reuse in the future to the main reviewer, two supervisors, and students.

### 2.3 Data items

Informed by the data charted, a full list and definition of all variables will be provided.

**Regarding data dissemination**, Results from this research will be shared on various platforms to reach relevant end users, policymakers, and service providers in the form of, for example, presentations at conferences, workshops, and publication of an article.

## 3 DISCUSSION

This section will discuss the data collected. In line with Tricco et al., (2016), this scoping review envisages outlining existing literature, identifying gaps, and informing future research in the field of self‐employment for persons with disabilities. *What evidence‐based self‐employment frameworks have been successfully used to promote self‐employment among persons with disabilities?* Is a question that this scoping review seeks to answer. To answer this research question, the objectives will be to identify and describe:

i. Existing self‐employment evidence‐based frameworks used with persons with disabilities
ii. Barriers and facilitators in using a self‐employment evidence‐based framework with persons with disabilities
iii. Types of successful self‐employment opportunities (small businesses) that persons with disabilities engage in

Thus, this research has the potential to add to knowledge and inform or stimulate further research. For instance, discussions from this scoping review are intended to inform future contextually relevant research.

This future research will be specific to occupational therapy and will entail consultations with relevant end users, service providers, and policymakers in the field of self‐employment from South Africa, KwaZulu Natal Province (Arksey & O'Malley, 2005). We envisage the end product in a form of a framework that includes but is not limited to, the purpose of the framework, how the framework was developed, user's guide, steps and processes to follow, checklists, funding and important contact details.

## 4 STRENGTH AND LIMITATIONS

### 4.1 Strength

Scoping reviews are cost‐effective and feasible. They allow the collection of international data across different professions to give a broader view of a topic of interest. A broader view may have the potential to benefit service providers, end users and policymakers. For instance, in the field of self‐employment:

‐ Service providers as beneficiaries: The findings may contribute to clinical practice such as informing curriculum taught in academia.
‐ End users as beneficiaries: Patients seen by multidisciplinary teams such as occupational therapists, industrial psychologists and social workers will also benefit in that they will have an alternative form of employment to generate income and improve their lives.
‐ Policymakers as beneficiaries: Such as the government, may benefit when its citizens become economically active and rely less on the government's, for example, disability grants.

### 4.2 Limitations

Due to the nature of this research, the following may be its limitations:

‐ Data in hard copy format, such as books, may be missed as this research will only consider online sources.
‐ Relevant data that is in non‐English may be missed as this study will only include English written data.

## AUTHORS' CONTRIBUTIONS

**Name**: Mr. Luther Lebogang Monareng

**Contribution**: Responsible for heading this project, conceptualising and draughting the protocol.

**Name**: Professor Shaheed Mogammad Soeker

**Contribution**: Assisted with the conceptualisation of the protocol, guidance and critical reviews throughout the writing process of the manuscript.

**Name**: Associate Professor Deshini Naidoo

**Contribution**: Assisted with the conceptualisation of the protocol, guidance and critical reviews throughout the writing process of the manuscript.

## COMPETING INTEREST STATEMENT

There are no known competing interests or conflicts associated with any of the authors and reviewers involved in this research.

## FUNDING

This work was supported by:

i. University Capacity Development Grant Funding (UCDP) from the University of KwaZulu Natal. The funding was used for the corresponding author's teaching relief while conducting this research.
ii. University of KwaZulu Natal's Step Up programme. The funding was used for the corresponding author's visit to the University College London for capacity building and mentorship.

## ETHICS APPROVAL STATEMENT

No ethics is required for this research as data will be collected from studies where the primary investigators have received informed consent and/or ethical approval. Moreover, there will be no animals and/or human participants involved in this study. Helsinki's declaration will also be upheld (Mundial, 2019).
